# Treatment outcomes and associated factors among patients with drug-resistant tuberculosis in Ethiopia: A retrospective cohort study

**DOI:** 10.1016/j.ijregi.2026.100905

**Published:** 2026-04-21

**Authors:** Abenezer Abraham Anito, Gemechis Abdissa, Yohannis Gezahegn, Ebrahim Umer Muhammed, Hunde Lemi

**Affiliations:** 1 Ethiopian Food and Drug Authority, Addis Ababa, Ethiopia; 2 BRIDGE Health Nexus, Addis Ababa, Ethiopia; 3 Department of internal medicine, Adama Hospital Medical College, Oromia, Ethiopia

**Keywords:** Drug-resistant tuberculosis, Treatment outcomes, Firth regression, HIV co-infection, Ethiopia

## Abstract

**Objectives:**

Drug-resistant tuberculosis (DR-TB) remains a major public health challenge globally. Despite diagnostic advances and expanded access to all-oral regimens in Ethiopia, treatment outcomes continue to vary across the country. Evidence on factors affecting treatment outcomes under programmatic setups is needed to inform targeted interventions. This study aimed to assess the treatment outcomes and associated factors of patients with DR-TB at Adama Hospital Medical College from January 2016 to December 2024.

**Methods:**

We conducted a retrospective cohort study among patients treated for DR-TB. Treatment outcomes were classified according to the World Health Organization definitions as successful (cured/completed) or unsuccessful (treatment failure/death/loss to follow-up). Sociodemographic, behavioral, and clinical characteristics were extracted from routine records using an online form administered via Kobo Toolbox. The data set was later exported in CSV format and analyzed using R statistical software (version 4.3.2). Descriptive statistics were used to summarize the characteristics of the study population. Bivariate logistic regression was initially performed. Variables with a *P* < 0.25 in the bivariate analysis were included into a multivariable Firth penalized logistic regression model addressing sparse data and separation to identify independent predictors of treatment outcomes. Statistical significance was determined at *P* < 0.05, with a 95% confidence interval (CI). Sensitivity analysis used a model controlling for collapsed regimen categories.

**Results:**

Among 249 eligible patients with MDR/RR-TB, 197 (79.1%) achieved successful treatment outcomes, whereas 27 (10.8%) died and 22 (8.9%) were lost to follow-up and three (1.2%) were treatment failures. Of the 249 patients included in the cohort, 238 were included in the multivariable analysis due to complete data availability. In the complete-case analysis using multivariable Firth model (n = 238), age ≥45 years was independently associated with an unsuccessful outcome (adjusted odds ratio [AOR] 3.43, 95% CI 1.23-9.89). Patients who were underweight at enrollment (AOR 2.47, 95% CI 1.19-5.36) had increased odds of unsuccessful treatment outcome. Previous defaulters (loss to follow-up) had substantially higher odds of unsuccessful outcome than new patients (AOR 7.13, 95% CI 1.02-84.26), although CIs were wide due to sparse data. Human immunodeficiency virus (HIV) co-infection was associated with increased odds of unsuccessful treatment outcome in the primary analysis (AOR 3.20, 95% CI 1.04-10.21). In the sensitivity analysis, the association remained in the same direction but with borderline significance (AOR 3.01, 95% CI 0.97-9.62).

**Conclusion:**

Despite high overall treatment success, older adults, undernourished, and previous defaulters remain at substantially increased risk of unsuccessful DR-TB treatment outcomes. HIV co-infection was also associated with unsuccessful treatment outcomes, with a directionally consistent association in sensitivity analysis. Targeted adherence support, strengthened TB/HIV integration, and improved programmatic monitoring are essential to further reduce mortality and loss to follow-up.

## Highlights

• Unsuccessful treatment outcomes occurred in 20.9% of patients with drug-resistant tuberculosis.
• Age 45 years or older was independently associated with poor treatment outcome.
• Underweight status increased the odds of unsuccessful outcomes.
• Previous loss to follow-up was strongly associated with unsuccessful outcome.
• Human immunodeficiency virus is associated with poor outcomes, with consistent effect in the sensitivity analysis.

## Introduction

Drug-resistant tuberculosis (DR-TB), encompassing rifampicin-resistant-TB (RR-TB) and multi-DR-TB (MDR-TB), remains a major barrier to global tuberculosis (TB) control. RR-TB is defined as resistance to rifampicin with or without resistance to other first-line drugs, whereas MDR-TB is defined as resistance to at least isoniazid and rifampicin. Despite advances in molecular diagnostics and expanded access to treatment, DR-TB continues to be associated with high mortality and suboptimal treatment success [1,2]. Worldwide, an estimated 390,000 MDR/RR-TB cases and 150,000 deaths were reported in 2024, although the incidence has remained relatively stable since 2020. Treatment success rates for DR-TB remain substantially lower that global targets, reflecting the complexity, toxicity, and prolonged duration of available regimens [3,4].

Low- and middle-income countries bear a disproportionate share of global DR-TB burden. Sub-Saharan Africa, hosting eight of the 30 high-burden countries, faces a dual epidemic of MDR/RR-TB and human immunodeficiency virus (HIV) that substantially complicates clinical management and drives mortality [2,5]. In Ethiopia, the burden of drug resistance poses a sustained threat to the national health security, despite the country’s transition out of the global list of 30 high MDR-TB burden countries in 2021. According to the 2024 World Health Organization (WHO) Global Tuberculosis Report, MDR/RR-TB) is estimated to occur in 1.1% of new TB cases and 12% of previously treated cases, reflecting a slight shift from earlier surveillance estimates [2,6,7]. Despite investments in diagnostic expansion and introduction of shorter and more effective regimens (e.g. BPaLM [bedaquillin + pretomanid + linezolid + moxifloxacillin]/BPaL [bedaquillin + pretomanid + linezolid]), treatment outcomes continue to vary widely across regions and facilities [[8], [9], [10], [11], [12], [13], [14]].

Improving treatment outcomes for DR-TB is central to the WHO End TB Strategy, which aims to reduce TB deaths by 95% and eliminate catastrophic costs by 2035 [15]. The introduction of harmonized WHO treatment outcome definitions in 2020 has improved comparability across studies and programmatic settings [16]. However, evidence from Ethiopia and other high-burden settings indicates that unfavorable treatment outcomes—including death, treatment failure, and loss to follow-up—remain common and are influenced by a complex interplay of demographic, clinical, and health system factors [11,12,14,[17], [18], [19], [20], [21], [22], [23], [24], [25], [26], [27], [28]].

Adama Hospital Medical College (AHMC) is a major DR-TB treatment initiation and referral center in Ethiopia, managing a substantial number of patients across different programmatic periods, including those before and after the introduction of newer all-oral regimens. Despite its clinical and programmatic importance, evidence on DR-TB treatment outcomes and their determinants from this setting is limited, with few programmatic analyses having applied bias-reduction methods to address sparse-event limitations. We aimed to evaluate the treatment outcomes among patients with DR-TB between 2016 and 2024 at AHMC and identify factors associated with unsuccessful outcomes using penalized regression methods, generating evidence to inform clinical practice and strengthen national DR-TB control efforts.

## Methods

### Study design

This was a single-center retrospective cohort study using routine programmatic DR-TB register data and clinical records.

### Setting

The study was conducted at AHMC, a tertiary referral hospital providing programmatic management of DR-TB through its Gada Treatment Initiation Center (TIC). Being one among other centers in the Oromia region for MDR/RR-TB care, it serves as a referral site for patients from neighboring health facilities. All eligible patients initiated on DR-TB treatment between January 2016 and December 2024 were included. Data abstraction was conducted from June to August 2025, using routinely collected clinical records and programmatic registers.

### Participants

Study participants were all patients diagnosed with MDR/RR-TB, were initiated on treatment at the AHMC Gada TIC between January 2016 and December 2024, and had a documented final treatment outcome recorded at the time of data extraction. Participants were identified from the hospital DR-TB treatment register. All consecutive eligible patients during the study period were included.

Inclusion criteria were laboratory-confirmed MDR/RR-TB based on drug sensitivity testing; initiation of MDR/RR-TB treatment at AHMC during the study period; completion of at least one MDR/RR-TB treatment regimen with a recorded outcome by the end of the study period; and availability of complete demographic, clinical, and treatment data.

Exclusion criteria were diagnosis of extensively DR-TB or other resistance patterns not meeting the MDR/RR-TB definition, cases diagnosed solely based on clinical decision without bacteriological confirmation, and records with missing treatment outcome data.

### Variables

#### Dependent variable

The dependent variable was MDR/RR-TB treatment outcome, dichotomized as successful (cured, completed treatment) vs unsuccessful (death, treatment failure, or lost to follow-up), according to the WHO definition [16].

#### Independent variables

Sociodemographic and behavioral factors included age, sex, residence, marital status, occupation, alcohol use, smoking, and Khat use.

Clinical factors included baseline body mass index (BMI), HIV status, presence of comorbidities, TB registration category (new or previously treated), baseline culture, baseline drug sensitivity test, site of DR-TB, baseline chest radiographic findings, treatment regime type, smear conversion, interim (6-month) culture result, and missed follow-up visits.

Status of HIV infection was ascertained from the routine registers as a documented serology test result. Anthropometric measurements were recorded at the time of enrollment. Radiologic evaluation using chest X-ray was done for most of the patients with pulmonary TB and extent of lung involvement was ascertained from baseline radiography reports.

Treatment regimens were categorized according to the WHO and National guidelines for MDR/TB treatment. The BPaLM regimen is a 6-month all-oral regimen for people with MDR/RR-TB (with or without resistance to fluoroquinolones). The short all-oral regimen represents several all-oral short regimens of 9 months for patients who do not have any resistance to fluoroquinolones. The long all-oral regimen represents longer regimens of 18-20 months that may include an injectable drug (amikacin). Individualized regimens are tailored regimens designed based on drug susceptibility testing, treatment history, or clinical considerations. The previous standardized regimen represents the previously used shorter 9-12–month injectable regimen.

### Data sources

Data were obtained through retrospective review of DR-TB registration books and individual patient medical records. A structured data extraction tool developed in English was deployed through Kobo Toolbox and used to collect information on sociodemographic characteristics, behavioral factors, clinical factors, and treatment outcomes. Data collection was conducted by trained BSc nurses with experience in MDR-TB care and the registration tools.

### Bias

The data extraction tool was pretested on 15 patient records at the study site, and refinements were made to improve clarity and consistency. Data collectors received a full-day training before the data collection on the standardized data abstraction tool, data collection procedures, techniques, and quality assurance. Completed extraction forms were submitted online and were audited for completeness, consistency, and quality by the principal investigator daily; any discrepancies were resolved through discussion. Potential selection bias was minimized through inclusion of consecutive eligible patients. Information bias was also reduced by using routinely collected standardized programmatic records.

### Sample size

A total population (census) approach was used. All eligible patients with MDR/RR-TB meeting the inclusion criteria within the specified period were included. No formal sample size calculation was performed.

### Quantitative variables

Age was categorized (<25, 25-44, and 45 years or older) based on epidemiologic relevance and previous literature. BMI was used to determine undernutrition based on WHO cutoff (<18.5 kg/m^2^).

### Statistical methods

The collected data were exported from the Kobo Toolbox in CSV format and analyzed using R statistical software (version 4.3.2). Descriptive statistics were used to summarize sociodemographic, behavioral, and clinical characteristics of participants, stratified by treatment outcome. Categorical variables were reported as frequencies and percentages, and continuous variables were summarized using medians and interquartile ranges. The primary outcome was treatment outcome, dichotomized as successful (cured or treatment completed) or unsuccessful (treatment failure, death, or loss to follow-up), in accordance with WHO definitions. Bivariate logistic regression was conducted to examine associations between independent variables and treatment outcomes. Variables with *P* < 0.25 in the bivariable analysis were included in the multivariable logistic regression model to control for confounding. Given the relatively small number of unsuccessful outcomes, evidence of sparse data, and quasi-complete separation for several predictors, multivariable Firth penalized logistic regression was used to reduce small-sample bias and provide finite, more stable estimates. Although the bivariate analysis was conducted using all available observations (n = 249), the multivariable Firth regression was restricted to complete cases (n = 238). Complete-case analysis was performed; missingness was <5% for all covariates. Adjusted odds ratios (AORs) with 95% confidence intervals (CIs) were reported, along with *P* values declared as significant when less than 0.05. Model performance was evaluated and revealed acceptable discrimination (C-statistic 0.75, 95% CI 0.67-0.83). Bootstrap internal validation (300 resamples) yielded a similar estimate (0.73-0.86), suggesting limited optimism. Multicollinearity was assessed using variance inflation factors.

### Ethical considerations

A waiver of informed consent was obtained from the institutional review board of AHMC. Permission to access patient records was granted by the Gada TIC administration. De-identified patient data were stored confidentially through password-protected mobile applications.

## Results

Between 2016 and 2024, 249 patients with DR-TB treated at the center met the inclusion criteria and were included in the overall cohort description. Among those, 11 (4.4%) participants were excluded from the multivariable analyses due to missing baseline covariate data, leaving 238 patients (49 events) in the complete-case analytic sample.

Among 238 patients included in the analysis, 189 (79.4%) achieved successful treatment outcomes (186 cured; three completed), whereas 49 (20.6%) had unsuccessful outcomes (26 deaths, 20 lost to follow-up, and three treatment failures).

The median age among the participants was 30 years (interquartile range 24-40; mean 32.5 [standard deviation (SD) 14.1]), comparable to the full cohort (mean 32.2 [SD 14]), male (69.4% vs 56.6%), HIV-positive (28.6% vs 15.3%), underweight (71.4% vs 48.7%), and had bilateral lung lesions (36.7% vs 18.8%) or previous loss to follow-up (8.2% vs 0.5%) upon registration. Longer regimens were also more common among patients with unsuccessful outcomes (44.9% vs 24.3%) (Table 1).

**Table 1:** Baseline characteristics of patients with DR-TB by treatment outcome at Adama Hospital Medical College, Ethiopia, 2016-2024 (N = 249)^a^.

Characteristic	Categories	Successful (n = 189), n (%)	Unsuccessful (n = 49), n (%)
Age group	25-44 years	95 (48.2)	20 (38.5)
	<25 years	71 (36.0)	17 (32.7)
	≥45 years	31 (15.7)	15 (28.8)
Sex	Female	82 (41.6)	15 (28.8)
	Male	115 (58.4)	37 (71.2)
Residency	Urban	86 (43.7)	28 (53.8)
	Rural	111 (56.3)	24 (46.2)
Smoking	No	169 (85.8)	39 (75.0)
	Yes	28 (14.2)	13 (25.0)
HIV status	Negative	167 (84.8)	38 (73.1)
	Positive	30 (15.2)	14 (26.9)
Underweight (BMI <18.5 kg/m²)	No	97 (49.2)	14 (26.9)
	Yes	100 (50.8)	38 (73.1)
Comorbidity	No	145 (73.6)	31 (59.6)
	Yes	52 (26.4)	21 (40.4)
Lung lesion extent	Unilateral	147 (77.8)	31 (63.3)
	Bilateral	42 (22.2)	18 (36.7)
Registration group	New	77 (39.1)	14 (26.9)
	Loss to follow-up	1 (0.5)	4 (7.7)
	Previous treatment failure	63 (32.0)	14 (26.9)
	Relapse	56 (28.4)	20 (38.5)
Regimen type	BPaLM	23 (11.7)	2 (3.8)
	Individualized	1 (0.5)	1 (1.9)
	Long all-oral regimen	48 (24.4)	23 (44.2)
	Short all-oral regimen	67 (34.0)	12 (23.1)
	Previous standardized regimen	58 (29.4)	14 (26.9)

Abbreviations: BMI, body mass index; BPaLM, bedaquillin + pretomanid + linezolid + moxifloxacillin; DR-TB, drug-resistant tuberculosis.

a Percentages are column percentages.

### Factors associated with treatment outcomes

In the bivariable logistic regression, patients aged ≥45 years (COR 2.30, 95% CI 1.04–5.03); sex (COR 1.76, 95% CI 0.92-3.50); HIV co-infection (COR 2.05, 95% CI 0.97-4.19)); underweight status (COR 2.63, 95% CI 1.37–5.31); Any comorbidity (COR 1.89, 95% CI 0.99-3.57)); bilateral lung lesions (COR 2.03, 95% CI 1.02–3.97); previous loss to follow-up (COR 22.00, 95% CI 2.99–447.96); patients receiving long all-oral regimens (COR 5.51, 95% CI 1.46–36.18) had p-values <0.25 and prioritized to be included in the multivariable model based on clinical relevance (Table 2).

**Table 2:** Bivariable logistic regression analysis of factors associated with unsuccessful treatment outcome among patients with DR-TB at Adama Hospital Medical College, Ethiopia, 2016-2024 (N = 249).

Variable	Category	Crude OR (95% CI)	*P* value
**Age group**	25-44 years	1.00 (reference)	—
	<25 years	1.14 (0.55-2.33)	0.725
	≥45 years	2.30 (1.04-5.03)	0.037^a^
**Sex**	Female	1.00 (reference)	—
	Male	1.76 (0.92-3.50)	0.095^a^
**Marital status**	Married	1.00 (reference)	—
	Single	0.86 (0.44-1.65)	0.660
	Divorced	1.00 (0.14-4.38)	1.000
	Widowed	0.39 (0.02-2.18)	0.379
**Residence**	Urban	1.00 (reference)	—
	Rural	0.66 (0.36-1.23)	0.191
**Smoking**	No	1.00 (reference)	—
	Yes	2.01 (0.93-4.18)	0.066
**HIV status**	Negative	1.00 (reference)	—
	Positive	2.05 (0.97-4.19)	0.052^a^
**Under**weight **(BMI <18.5 kg/m²)**	No	1.00 (reference)	—
	Yes	2.63 (1.37-5.31)	0.005^a^
**Anemia**	No	1.00 (reference)	—
	Yes	2.32 (0.94-7.02)	0.094
**Any comorbidity**	No	1.00 (reference)	—
	Yes	1.89 (0.99-3.57)	0.051^a^
**Lung lesion extent**	Unilateral/minimal	1.00 (reference)	—
	Bilateral	2.03 (1.02-3.97)	0.039^a^
**Cavitary disease**	No	1.00 (reference)	—
	Yes	0.93 (0.46-1.80)	0.824
**Registration group**	New case	1.00 (reference)	—
	Previous loss to follow-up	22.00 (2.99-447.96)	0.007^a^
	Previous treatment failure	1.22 (0.54-2.77)	0.628
	Relapse	1.96 (0.92-4.29)	0.084
**Regimen type**	BPaLM	1.00 (reference)	—
	Individualized	11.50 (0.36-389.41)	0.126
	Long all-oral regimen	5.51 (1.46-36.18)	0.029^a^
	Short all-oral regimen	2.06 (0.51-13.87)	0.367
	Previous standardized	2.78 (0.70-18.56)	0.199

Abbreviations: BMI, body mass index; BPaLM, bedaquillin + pretomanid + linezolid + moxifloxacillin; CI, confidence interval; DR-TB, drug-resistant tuberculosis; OR, odds ratio

a Significant variables at p<0.25 prioritized based on clinical relevance

In the multivariable penalized logistic regression (including 238 participants with 49 unsuccessful outcomes), older age (≥45 years), underweight status, previous loss to follow-up, and HIV co-infection were independently associated with unsuccessful treatment outcomes in patients with DR-TB. Adjusted estimates were derived from a multivariable Firth logistic regression model including, age, sex, HIV status, BMI, comorbidity, lung lesion extent, registration group, and regimen type (Table 3).

**Table 3:** Multivariable Firth penalized logistic regression of factors associated with treatment outcome among patients with DR-TB at Adama Hospital Medical College, Ethiopia, 2016-2024 (N = 238).

Characteristics	Categories	Adjusted OR (95% CI)	*P* value
**Age group**	25-44 years	1.00 (reference)	—
	<25 years	1.42 (0.60-3.34)	0.423
	≥45 years	3.43 (1.23-9.89)	0.019^a^
**Sex**	Female	1.00 (reference)	—
	Male	1.62 (0.78-3.50)	0.194
**HIV status**	Negative	1.00 (reference)	—
	Positive	3.20 (1.04-10.21)	0.043^a^
**Underweight (BMI <18.5 kg/m²)**	No	1.00 (reference)	—
	Yes	2.47 (1.19-5.36)	0.014^a^
**Any comorbidity**	No	1.00 (reference)	—
	Yes	0.68 (0.23-1.90)	0.461
**Lung lesion extent**	Unilateral/minimal	1.00 (reference)	—
	Bilateral	1.56 (0.69-3.45)	0.281
**Registration group**	New case	1.00 (reference)	—
	Loss to follow-up	7.13 (1.02-84.26)	0.048^a^
	Previous treatment failure	0.93 (0.36-2.33)	0.876
	Relapse	1.45 (0.61-3.50)	0.398
**Regimen type**	BPaLM	1.00 (reference)	—
	Individualized	11.87 (0.70-219.87)	0.083
	Long all-oral regimen	3.40 (0.83-20.00)	0.092
	Short all-oral regimen	1.79 (0.43-10.54)	0.443
	Previous standardized regimen	2.14 (0.49-13.08)	0.327

Abbreviations: BMI, body mass index; BPaLM, bedaquillin + pretomanid + linezolid + moxifloxacillin; CI, confidence interval; DR-TB, drug-resistant tuberculosis; OR, odds ratio

a Significant at p<0.05

Compared with patients aged 25-44 years, those aged ≥45 years had more than three-fold higher odds of unsuccessful outcome (AOR 3.43, 95% CI 1.23-9.89). Patients with HIV co-infection had increased odds of unsuccessful treatment outcome (AOR 3.20, 95% CI 1.04-10.21), as did patients who were underweight at baseline (AOR 2.47, 95% CI 1.19-5.36). Patients previously lost to follow-up had substantially higher odds of unsuccessful outcome than new patients (AOR 7.13, 95% CI 1.02-84.26), although CIs were wide due to sparse data (Table 3).

Sex, comorbidity status, extent lung involvement, and regimen type were not independently associated with outcome after adjustment.

Sensitivity analyses were conducted collapsing categories and excluding regimen categories that showed consistent associations for older age, underweight status, and registration as previous defaulter (loss to follow-up), whereas HIV co-infection, although directionally consistent, remained of borderline significance (Supplementary Table S1).

## Discussion

In our analysis of this single-center program-enrolled patients with MDR/RR-TB, the overall treatment success rate was 79.1%, comparable to the complete-case analytic sample (79.4%; 189 of 238). Unsuccessful outcomes were primarily as a result of death (51.9%) and loss to follow-up (42.3%), with a small minority (5.8%) accounted for by treatment failure. This pattern, where mortality and attrition contribute more to unsuccessful outcomes than treatment failure, is similar to reports from other programmatic data globally and regionally, where early death and disengagement from care remain the dominant contributors of unsuccessful outcome despite advances in regimen potency and duration [8,[11], [12], [13],^21^]. In settings with intensive follow-up where loss to follow-up was significantly reduced and with higher diagnostic capacity, treatment failure was reported as the highest contributor for poor treatment outcomes in DR-TB treatment [21]. In contrast to our findings, another report from our country has indicated that with short-term injectable regimen for MDR-TB, the highest contributor to poor outcomes was treatment failure. This could have been due to the drug resistance patterns and regimen complexity in this particular population [13].

In the multivariable Firth regression, older age (45 years or older), HIV co-infection, undernutrition, and previous loss to follow-up upon registration were independently associated with unsuccessful treatment outcomes. Our findings revealed three-fold higher odds of unsuccessful outcome among older patients similar to national Ethiopian analyses and global reports linking poor outcomes with advanced age [11,12,21,22,25,27]. Studies have also shown increased odds of death for age ≥45 to 60 years [11,12,21,25,27]. This is most likely from immunosuppression, comorbidities, and delayed care seeking.

Low BMI, a proxy for malnutrition, demonstrated a strong association with unsuccessful treatment underscoring the well-established relationship between malnutrition and disease progression, treatment intolerance, and immunosuppression [8,10,14,27]. This factor is widely explored in multiple studies in Ethiopia and, as such, supports integrating nutritional assessment and supplementation into DR-TB care pathways.

Patients enrolled after the previous default as loss to follow-up had markedly elevated odds of unsuccessful outcomes, although with wide CIs. Although loss to follow-up was not explored much as a predictor, evidence suggests previous treatment, socio-economic hardship, and programmatic barriers associated with high loss to follow-up, suggesting a persistent effect across treatment episodes [8,12,19,21].

Although the rate of loss to follow-up seems to be lower in our setting, the significant association with poor outcome in future treatment episodes has programmatic implications underscoring adherence support.

Our findings also show that co-infection with HIV is independently associated with poor outcomes in the primary model. Although the association was only borderline significant in the sensitivity analysis, the direction of effect remained consistent. This is in line with previous literature that showed TB/HIV co-infection increases the odds of poor outcomes and/or death even in the era of antiretroviral therapy [8,10,11,14,20,25] The three-fold higher odds of unsuccessful outcome in our study is in line with other similar studies in our setting and other countries, which also have shown a two to five times higher risk of death, a major contributor among poor outcomes [8,10,11,14,20].

We also want to highlight that although regimen type was not independently associated with outcome after adjustment, longer regimens showed a non-significant trend toward unsuccessful outcomes. Descriptive data also demonstrated a higher proportion of patients on longer regimens with unsuccessful outcomes. This could have likely been a reflection of possible confounding by indication—patients with more extensive disease or resistance profiles preferentially being given longer or individualized regimens.

With the programmatic introduction of 6-month BPaL/BPaLM regimens in Ethiopia, continued monitoring would be much needed to determine whether shorter, all-oral regimens translate into sustained improvements in survival and retention under routine conditions.

Notably, disease severity markers such as bilateral lung disease and comorbidity lost significance after adjustment, possibly suggesting mediation of effects through nutritional status, HIV infection, or age. The use of Firth penalized logistic regression mitigated small-sample size bias given the limited number of events (n = 49), and effects estimated were robust across sensitivity analyses, strengthening confidence in the identified factors.

In general, our findings reinforce that achieving End TB strategic targets for DR-TB in our country requires more than optimized regime alone. Integrated TB/HIV services, aggressive nutritional support and timely management of malnutrition, early identification of high-risk older patients, and intensive adherence interventions in individuals with previous treatment history are critical to reducing unsuccessful outcomes, death in particular, among patients with DR-TB.

The findings of this study should be interpreted in light of the limitations, including possible imprecision of association, especially for the registration group, as seen from wider CIs; the design also limits causal inference.

## Declaration of competing interest

The authors have no competing interests to declare.

## Appendix Supplementary materials

Image, application 1
