# Supplementary material for: Treatment outcomes and associated factors among patients with drug-resistant tuberculosis in Ethiopia: A retrospective cohort study
Source: IJID Reg. 2026 Apr 21;19:100905. doi: 10.1016/j.ijregi.2026.100905 (PMC13202542; doi:10.1016/j.ijregi.2026.100905)
Supplement: Supplementary file 1 [file mmc1.docx]

# Sensitivity Analysis

A sensitivity analysis using collapsed binary regimen variables (longer Vs shorter) was conducted using a Firth penalised logistic regression model, to assess the robustness of the primary model and address sparse-category bias related to regimen classification. When categorizing the regimens “BPaLM”, and “Short all-oral regimen” were categorized as shorter, while “Long all-oral regimen”, "Individualized regimen", and “previous standardized regimen” were categorized as longer regimens.

The results of this analysis have confirmed that the direction and magnitude of associations remained consistent with the primary model. Older age, underweight status, and registration as previous defaulter (loss to follow-up) remained independently associated with unsuccessful treatment outcome. Co-infection with HIV remained of borderline significance (AOR 3.01, 95% CI 0.97-9.62). Regimen type was not independently associated with treatment outcome. ([Supplementary Table S1](#36t79t8f3zkx))

These findings indicate that the primary inferences were robust.

###

### Supplementary Table S1. Firth penalised logistic regression sensitivity analysis with collapsed regimen variable

| **Variable** | **Adjusted OR (95% CI)** | **p value** |
| --- | --- | --- |
| **Age group** | | |
| 25–44 years | 1.00 (reference) | — |
| <25 years | 1.40 (0.61–3.23) | 0.427 |
| ≥45 years | 3.35 (1.21–9.54) | 0.020 |
| **Sex (male)** | 1.81 (0.89–3.84) | 0.102 |
| **HIV positive** | 3.01 (0.97–9.62) | 0.056 |
| **Underweight** | 2.47 (1.20–5.33) | 0.013 |
| **Comorbidity** | 0.69 (0.23–1.94) | 0.487 |
| **Bilateral lung lesion** | 1.43 (0.65–3.08) | 0.375 |
| **Registration group** | | |
| New case | 1.00 (reference) | — |
| Loss to follow-up | 9.16 (1.36–105.61) | 0.022 |
| Previous treatment failure | 0.97 (0.39–2.40) | 0.951 |
| Relapse | 1.73 (0.75–4.06) | 0.196 |
| **Longer regimen (binary)** | 1.87 (0.90–4.04) | 0.093 |
